# Loot

**Published:** 1873-10

**Authors:** 


### Loot.

***Treatment of Traumatic Neuralgia.*** By S. Weir Mitchell, Physician to the Philadelphia Orthopœdic Hospital and Infirmary for Diseases of the Nervous System. ("Injuries of Nerves, and their Consequences,") 8vo., p. 337. London: Trubner & Co. 1872.

In the treatment of traumatic neuralgia the author says:

"The pains of traumatic neuralgia are so terrible that we are usually driven at once to the use of narcotic hypodermic injections, without which it would often be impossible to relieve such cases. In neuralgia, from what we call, for want of a better term, irritation of the nerves, there is reason to believe that some of the opiates, in the form of hypodermic injection, may prove more or less curative in their action; but where, as in most traumatic neuralgias, there is manifest organic alteration of the nerve, such agents are chiefly of service, because they relieve pain, and thus enable us to bridge over, so to speak, the many months of torture which are needed to bring the nerve back to health again, or to afford time for electrical or other treatment. * * * At the present time, this mode of using narcotics has grown into common use, but even yet it is scarcely estimated at its full value. In the wards for nerves and wounds in the U. S. A. Hospital, it is almost the only plan of treating severe neuralgic pain; so that twice or thrice a day the resident surgeons pass around these wards with their narcotics and hypodermic syringes, seeing, as a physician observed to me, anguished and troubled faces before them, and leaving behind them comfort, and even smiles.

"During one year at least forty thousand doses of various narcotics were administered without an accident, and in certain single cases upwards of five hundred hypodermic injections were used; so that, if there were no other evidence of the innocence of this mode of medication, our own experience would have been amply competent to settle the question. I have had large opportunity of studying, in traumatic neuralgia, the comparative merits of narcotics used by the mouth or under the skin, and I have no hesitation in stating my opinion, that the latter method is not only the more effective, but also the less harmful, constitutionally. For the easing of neuro-traumatic pain we tried, in turn, the whole range of medicines known as narcotics, such as conia, hyoscyamus, daturia, atropia, and morphia. None of them, save the last, seemed, when singly used, to be of the slightest value; and, one by one, they were laid aside, until in the vast mass of cases, the salts of morphia alone were employed. A careful series of examinations showed, very distinctly, the trifling influence of atropia upon this form of pain. Several cases of intense neuralgia from wounds were treated with injections of sulphate of morphia under the skin in rising doses. When we learned the amount needed to give entire ease, we used in its place next day a full dose of atropia, our largest injection having been one-fifteenth of a grain. The most absolute failures attended these efforts; so that without denying to this latter drug the power ascribed to it of relieving certain neuralgias, I am sure that it is in the traumatic species simply useless. The morphia salts, on the other hand, are invaluable. For hypodermic use, I usually employ at first one-fourth of a grain of the sulphate, but I have given as much as a grain and a half twice a day. When continuously used, it is curious that its hypnotic manifestations lessen, while its power to abolish pain continues; so that the patient who receives a half grain or more of morphia may become presently free from pain, and yet walk about with little or no desire to sleep. The ability to lessen pain is not, therefore, of necessity, connected with the sleep-compelling potency. Where, however, the latter is inconveniently felt, and we desire the former only, it is possible to attain the end in view by using with the morphia a certain share of atropia. Thus, if we inject half a grain of sulphate of morphia, and with it the thirtieth of a grain of sulphate of atropia, the anæsthetic force of the morphia will remain unaltered, but the tendency to sleep will be greatly diminished. The views here set forth were reached after long and careful experiments on large numbers of men, and seem to justify the practice of using atropia and morphia together. As regards the place of injection, I agree with most observers that it is generally of little moment, the effect being the same whether it be thrown into the affected limb or into a remote part."—*Half-Yearly Abstract*, *Med. Sciences*, 1873.

***Mercury and Iodide of Potassium in Syphilis.***

Dr. Willard Parker of New York, in a recent clinical lecture reported in the *Medical Record*, sets forth the following views:

"I am aware that I differ with many of my brethren in the treatment of syphilis, but I believe that the poison of syphilis can only be removed from the system in almost all cases by the judicious use of mercury. This mercury is to be used wisely and in moderate doses, so as not to impair the vigor and health of the system. Very often it is important to make use of some tonic at the same time, such as quinine or the preparations of bark. These have been my convictions for a great many years, and I give them as the result of my own practical observation, and have never seen any reason to vary the conviction that iodide of potassium, alone, cannot overcome the syphilitic poison in the system. The iodide of potassium, however, is a very valuable remedy in the treatment of syphilis, but it comes in after we have accomplished our purpose with mercury, in order to remove any deleterious effects of the mercury which may be left in the system. Here its value cannot be over-estimated. The powerful effect which the iodide of potassium has upon the system, especially where mercury has been employed pretty freely, is sometimes seen in the profuse ptyalism which it produces, and if the syphilis receives any benefit from the administration of iodide of potassium, I believe it is in those cases which have been heretofore treated with mercury, and the iodide arouses the mercury to new action. You can remove mercury from the system by the use of iodide of potassium, but you can never remove syphilis by using it. At the same time that we use iodide of potassium in order to get good results in the system, I almost always employ the iodide of iron, as you see in this case. The point is, as has been stated, to bring the system up to par. The usual formula which I employ consists of six drachms of the iodide of potassium, one ounce of syrup iodide of iron, and make a six or eight ounce mixture. * * * The plan which I adopt and recommend in the treatment of syphilis is as follows: Take a case of genuine Hunterian chancre. I commence with the administration of iodide of mercury in one-half grain doses twice in twenty-four hours, combined with something, perhaps hyoscyamus or lactucarium, to prevent irritation of the mucous membrane of the intestinal canal. Continue this, in connection with a true diet, consisting of simple plain material and such as will produce healthy blood, embracing breadstuffs, eggs, milk, and meat twice a day, and cutting off entirely tobacco and all alcoholic drinks; continue the doses until the feeling of hardness about the chancre is all gone. Then stop the remedy, and watch the patient. If the disease begins to come out in the system, manifesting itself by glandular enlargements, diseases of the skin, affections of the fauces, or any one of these evidences, which shows that the poison is still in the system, resume the mercury as before and continue it until the disease has again passed away. It will be necessary to watch these patients for a long time, at least for months, and perhaps for a couple of years or more."

### ***Capital Punishment.***

We will doubtless all unite in the opinion that certainty of execution is the chief value of all penalty. This *uncertainty*, or rather almost absolute certainty that the extreme penalty will not be inflicted, is an encouragement to the murderer that his crime will somehow go unpunished. These questions of criminal law, however, are not so much a matter of professional inquiry or interest as that other question—how shall the extreme penalty of the law be best fulfilled? The question of humanity should never interfere with the power and influence of law; but if the control of law and the sanctity of humanity can be made to accord, all will certainly say 'amen!' If it be well to make death the penalty of certain crimes, the question comes to medical men, how shall this extreme punishment be inflicted so as to meet the requirements of justice, and yet avoid unnecessary cruelty in its execution. In various countries, death has been secured by hanging—as in this country—by the garrote, by the guillotine, by shooting. Some experiments by Prof. Brown-Sequard would seem to indicate that death by the guillotine is perhaps one of the most cruel of all known modes. Of course a headless man does not come back to tell us how long or how extremely he suffered; but we are led to infer that the cruelty of this punishment is beyond the general conception. Death by hanging is, to say the least of it, coarse and brutal. Its influence on the public mind cannot be elevating or conservative of morals of itself. So we come back to repeat, *extreme* punishment, as by death, is only of value, so far as penalty follows closely on crime. Now, if death shall continue to be a proper penalty for murder, the question for us, as medical men, to consider, is, how shall death be inflicted? Shall we shoot the culprit, or hang him like a dog, or cut off his head? The displays to morbid appetite in first-class hangings are too notorious to require comment. We respectfully suggest, then, that our professional influence should be exerted in favor of a more rational and decent death penalty. Let the criminal be required to die by chloroform, for example, or the inhalation of carbonic acid gas, quietly, in his cell, surrounded, if you please, by his medical and spiritual advisers. Death by chloroform is sufficiently certain; should not be a matter of public and morbid exhibition; is not cruel; all the requirements of the law are complied with. We demand, then, these two points—certainty of execution and humanity of the manner.—*Cincinnati Lancet and Observer.*

***Imposition on Life Insurance.***—*A New Department of Medical Journalism. An Interview between a Medical Journal Canvasser and a Medical Officer of a Life Ins. Co.*

Ques. "Doctor, I have called to present you with a copy of our *Medical Journal*, and call your attention to the new branch of medical science we have lately introduced. Don't you think the subject of Life Insurance a very important one to bring before the medical profession?" Ans. "Certainly." Ques. "Well, you see, we call it the 'Medical Department of Life Insurance.' We solicit from the medical officers of all the Life Insurance Companies articles bearing upon this subject, and would like to have you furnish us one for our next number. We wish to make this department instructive, especially for the benefit of medical examiners throughout the country, and it will be a very cheap way for Life Insurance Companies to advertise and furnish such kind of information. Several of the larger companies have already agreed to take several hundred copies to send to their examiners, as we can furnish it to them at a much reduced cost from the regular subscription price." Ans. "Ah, I see; then the whole gist of the matter is, that your publishers propose, by extracting what little phosphoric elements may be squeezed from the brains of the companies' medical officers, without compensation, to illuminate their rustic brethren in the same line, and thus create a demand for new subscribers. In this way you may 'hit two birds with one stone.' Well, this may be all very well for increasing publishers' incomes, but don't you think it is a little like 'crowding the mourners'?

"Now, I pay the full subscription price for your Journal, and you propose to add to your income by *peddling to* companies your Journal at half cost, at my and my medical brethren's expense. You wish to get valuable articles without cost, then induce companies to subscribe for a large number of copies at reduced prices, to circulate among their medical examiners, to induce these throughout the country to become subscribers, to make your Journal a pecuniary success, and also advertise your other published works.

"It really seems to me that the medical profession are already pretty well taxed, physically and mentally, in the various walks of this life. With gratuitous hospital and private services rendered the poor and needy, uncollectable bills, with income barely adequate for supplying themselves with the necessaries of life, to say nothing of numerous ephemeral medical journals, now to be called upon to contribute their 'mite' to swell the coffers of large publishing houses, by voluntary contributions, is asking a 'leetle too much.' I have always admired that trite old saying of Scripture, 'The laborer is worthy of his hire,' and in this age of 'strikes,' think the medical profession should join in refusing to work any longer for cormorant capital, without adequate compensation. I hope to see the day when medical journalism will be placed upon the proper business relation that daily journals are,—that its corps of contributors shall be compensated for their time and labor. Then we would see articles having true merit, full of instruction, and from the most learned in the profession, instead of the *advertising squibs* which now fill them. Don't you think the National Medical Association ought to interdict, in the code of ethics, publishing in medical journals articles with their authors' names and titles as specialists? If a medical gentleman has true merit in any special branch, his peers will soon find it out, without resorting to such doubtful means to catch the public eye."—*The Sanitarium.*

***Life Insurance.***

I am much gratified to see that you have added the medical side of life insurance to the many other interesting and practical features of your journal. The articles presented are all of much practical value, and just this kind of information is sadly needed by the majority of medical examiners. Permit me, as an examiner of some experience, to call attention to some points connected with the business, which I think deserve attention.

The article in the *Record* for May 15, entitled "On the Relation of the Medical Examiner to the Business of Life Insurance," by a Secretary, is very just and true, and certainly demands no more of examiners than a proper personal independence and appreciation of professional obligation would at all times prompt the *right-minded* to perform. But just here is the trouble, and the secretary evidently understands it, for he says it is this very want of personal independence which is far more dreaded by underwriters than any mere want of professional capacity. Unfortunately the two combined are not unfrequently met with, and I have seen and known of more than a few instances of such an utter want of both in the examiner as to make him the *subservient tool* of the agent. It is the agent's business to solicit risks, and it is his pecuniary interest to have those risks approved at the home office; so he is never backward in using an ass of a doctor if he can. And not unfrequently does it occur that the agent purposely avoids giving business to a man he knows to be competent and independent, preferring a more *compliant* examiner.

I have been cognizant of this state of things for so long a time, and have seen but little, and very imperfect, effort made for its correction, that I have begun to think the companies were indifferent about it—supposed they were making fortunes rapidly under this kind of procedure, and did not care to make any additional effort to correct abuses. If a remedy for it should be sought, I would suggest that one step in the right direction has been taken in securing the co-operation of your journal; another, and most prompt and decided check on these abuses, would be the securing of *competent and reliable medical men* as inspectors of agencies, paying such salaries as would command competent men, who would give the business their entire time. A competent medical man would, by the very nature of his professional training, and sharpened powers of observation, be capable of going into any agent's district, making the acquaintance of the physicians acting as examiners, and *others not so acting*, and render a far more correct opinion, *cæteris paribus*, than a non-professional inspector.

Permit me, in closing, to suggest that according to my observation here in the South, the tables of average weight to height are at least ten pounds higher than a correct estimate. MED. EXAM.

—*Medical Record.*

